# Revisiting the classics on secularization theory

**DOI:** 10.3389/fsoc.2025.1635582

**Published:** 2025-07-16

**Authors:** Haldun Gülalp

**Affiliations:** Independent Researcher, Istanbul, Türkiye

**Keywords:** secularization theory, sociology of religion, Max Weber, Karl Marx, Emile Durkheim

## Abstract

Secularization theory's sway has waned since the end of the 20^th^ century, but the myth that the “founding fathers” of sociology were pioneers of this theory has survived. This article aims to demolish this myth through a comparative analysis of the works of Karl Marx, Max Weber, and Emile Durkheim, and raises fresh questions about the concept of secularization.

## Introduction

Secularization theory is succinctly defined by its proponents as the assertion “that the social significance of religion diminishes in response to the operation of three salient features of modernization, namely (1) social differentiation, (2) societalization, and (3) rationalization” (Wallis and Bruce, 1992, p. 8–9; see also Tschannen, 1991). This theory, to be clearly distinguished from the normative political principle of secularism (Gülalp, 2022, 2023), became prominent in the immediate postwar era as a concomitant of modernization theory, but began to decline in the late 1970s and early 1980s with the global spread of religious politics. Observing the Islamic revolution in Iran in 1979, the Catholic liberation theology-inspired Sandinista revolution in Nicaragua in the same year, the collaboration between the Solidarity movement of the working class and the anti-communist Catholic Church in Poland in the 1980s, and the contemporaneous rise of the Christian right in the US and elsewhere, scholars came to acknowledge the failure of the theory's predictions (e.g., Douglas, 1982). After the end of the Cold War, the theory was declared dead and buried (Wuthnow, 1991; Stark, 1999), even if the search continued for possible ways to reformulate or salvage parts of it (Gorski and Altinordu, 2008).

In the received wisdom of the age of modernization theory, classical sociologists too were considered secularization theorists. Some devotees of the formerly popular theory still hold this view. Pippa Norris and Ronald Inglehart, for example, begin their widely-read book, *Sacred and Secular*, with the following words: “The seminal social thinkers of the nineteenth century—Auguste Comte, Herbert Spencer, Émile Durkheim, Max Weber, Karl Marx, and Sigmund Freud—all believed that religion would gradually fade in importance and cease to be significant with the advent of industrial society” (Norris and Inglehart, 2011; p. 3). This apparently standard formula may also be found in the works of other researchers, including those who are critical of the theory (e.g., Fokas, 2011; Clark, 2012).

I submit that particularly the three “founding fathers” of sociology could not be characterized as secularization theorists—except perhaps Max Weber and only to the extent allowed by the ambiguities in his writings. It is much easier to refute the notion for Karl Marx and Emile Durkheim. There is also a remarkable similarity between the views of these two theorists on the origins of religion, but a fundamental difference on the *possibility* of its decline. We may incidentally note that the term “secularization” does not appear in the works of any of these thinkers (or does so only rarely). Although they may have been secular (non-religious), each in his own way, there is no indication that they share the predictions of the 20^th^-century secularization theory.

## Weber on rationalization

A theory of secularization cannot be derived from Max Weber's *The Protestant Ethic and the Spirit of Capitalism* (1905). Although variously interpreted, this book's central argument is neither that Protestantism (leading subsequently to secularization) was an outcome of capitalist development (or modernization), as per the standard secularization theory, nor that it caused the rise of capitalism, in a presumed effort to invert the Marxian materialist causation. Rather, the book argues that capitalism as a socio-economic system and Protestantism as a belief system were in a relationship of “elective affinity,” where neither one caused the other but both had their common origins in rationalism (Löwith, 1982; Turner, 1985; McKinnon, 2010). In the conclusion of the book (Weber 1930, p. 183) states, “it is, of course, not my aim to substitute for a one-sided materialistic an equally one-sided spiritualistic causal interpretation of culture and history.” He also explains in the chapter on “Religious Groups” in *Economy and Society* (1920) that “religion nowhere creates certain economic conditions unless there are also present in the existing relationships and constellations of interests certain possibilities of, or even powerful drives toward, such an economic transformation.” (Weber, 1968, p. 577).

Weber's just cited essay on “Religious Groups” (also published separately as Weber, 1963) does not contain a theory of secularization either, although he alludes to the “rationalization” of religion, by which he means its growth out of and eventual separation from magic (Weber, 1968, p. 399–634). The concept of “rationalization,” a central theme in Weber's works more generally, which he uses liberally and imprecisely with occasional inconsistency, has a specific meaning in this context. Weber suggests that “the two characteristic elements of divine worship, prayer and sacrifice, have their origins in magic”; but, he further argues, in the course of religion's evolution “the belief in spirits became rationalized into belief in gods” (Weber, 1968, p. 422, p. 437). In this way, he claims, belief acquired “some measure of doctrine [as] the distinctive differential of prophecy and priestly religion, in contrast to pure magic” (Weber, 1968, p. 563). But Weber also repeatedly emphasizes that the separation from magic is never complete, “not even in Christianity” (Weber, 1968, p. 419–420, *passim*). Thus, “rationalization” in this context does not refer to “secularization” as defined above, but to the separation of religion from magic.

A theory of secularization is sometimes mistakenly discovered in Weber's concept of “disenchantment,” a term that he seldom uses and again with various meanings. It is often, yet controversially (Etzrodt, 2024; Marotta, 2024; Yelle and Trein, 2021; Jenkins, 2000), seen as a central element of his theory and deemed to imply that scientific progress would displace religious belief. The presumed source of this idea in Weber's work is his essay on “Science as a Vocation” (1918), wherein he repeatedly refers to scientific progress as the cause of “increasing intellectualization and rationalization,” which in turn “means that the world is disenchanted” (Weber, 1946a, p. 139). He says: “One need no longer have recourse to magical means in order to master or implore the spirits, as did the savage, for whom such mysterious powers existed. Technical means and calculations perform the service. This above all is what intellectualization means” (Weber, 1946a, p. 139). Clearly, for Weber, science would displace religion if it were still a type of magic, but it no longer is (despite some ambiguity).

Apropos this point, Philip Gorski describes the Protestant Reformation in Weberian terms as follows: In the “Middle Ages … faith and magic were not so much opposed as intermingled.” … “What occurred [during the Reformation] … is a rationalization of religion” (Gorski, 2000, p. 139, p. 148). The Protestant Reformation is often considered the origin of secularization, because it created the concept of “religion” as a distinct domain of social life along with the “secular,” in a political process whereby the territorial state established its hegemony at the expense of religion (see, e.g., Asad, 2003). But the Protestant movement was opposed to the worldly power and corruption of the Catholic Church and not to religion or religiosity. It affirmed that religious belief was a matter of a personal bond between God and the individual believer, and the text of the Bible was of paramount importance.

The Reformation(s) may have laid the foundation for pluralizing and eventually privatizing confessions, but it did not necessarily lead to a decline in religious faith in general and it did not even lead to the secularization of state power. Gorski notes that in the realm of social and cultural life, there occurred a deepening of “Christianization,” i.e., a movement away from magic, superstition, and folk religion, which was widespread among the people *and* the clergy; and “on the political level, … a deepening of the alliance between church and state.” This means that “if secularization is defined as the differentiation of religious and non-religious roles and institutions, the centuries after the Reformation can actually be seen as an era of radical *de*-secularization” (Gorski, 2000, p. 152, p. 159).

Weber argues in *Economy and Society* that scientific progress contributed to the evolution (or rationalization) of religion by helping its separation from magic: “As intellectualism suppresses belief in magic, the world's processes become disenchanted, lose their magical significance, and henceforth simply ‘are' and ‘happen' but no longer signify anything.” (Weber, 1968, p. 506). In a complementary statement in “Science as a Vocation,” he observes that “science today is irreligious” (Weber, 1946a, p. 142), which does not mean, as is sometimes thought, that science is (necessarily) opposed to religion, but that science and religion deal with *different* kinds of questions. He then asks if it can “be proved that the existence of the world which these sciences describe is worthwhile, that it has any ‘meaning,' or that it makes sense to live in such a world,” and replies, “Science does not ask for the answers to such questions” (Weber, 1946a, p. 144).

Returning to the topic of disenchantment toward the end of the essay, he states more generally: “The fate of our times is characterized by rationalization and intellectualization and, above all, by the ‘disenchantment of the world.' Precisely the ultimate and most sublime values have retreated from public life either into the transcendental realm of mystic life or into the brotherliness of direct and personal human relations” (Weber, 1946a, p. 155). This also applies to scientists themselves: “science ‘free from presuppositions' expects from [the scientist that] … if the process can be explained without those supernatural interventions, … [then it] has to be explained [that way] … And the believer can do this without being disloyal to his faith” (Weber, 1946a, p. 147). We may therefore conclude along with Etzrodt (2024, p. 658) that “disenchantment simply referred to the elimination of magic in the religious context” and with Jenkins (2000, p. 19) that “Secularization and disenchantment are not the same things, although they are easily confused.”

Finally, a theory of secularization is sometimes derived from Weber's writings by attributing to him a concept of “social differentiation” (a term that Weber does not actually use), that is, the autonomization of distinct spheres of social life caused by modernization, leading to a tension with religion (e.g., Casanova, 1994, p. 17–25; see also the literature cited in Eastwood and Prevalakis, 2010, p. 90–91). It is true that in “Religious Rejections of the World and their Directions” (1915) Weber speaks of possible “tensions” between religion(s) on the one hand and the economic, political, and other orders (such as the esthetic or erotic “spheres”) on the other. But he also indicates ways in which modes of accommodation or “escape” have been found (Weber, 1946b). (Hughey 1979, p. 107), for instance, after refuting other variants of secularization theory derived from Weber, lays emphasis on Weber's notion of parallel and competing rationalizations between these spheres, specifically economic and political spheres, and notes that it is “only in terms of this broad historical trend of differential institutional development, intellectual rationalization and compromise that ‘secularization' can mean anything at all.” Weber indeed notes, “The mutual strangeness of religion and politics, when they are both completely rationalized, is all the more the case…” (Weber, 1946b, p. 335).

But Hughey's interpretation is problematic because Weber's concept of rationalization is rather imprecise. As we saw above, and as (Hughey 1979, p. 101) elaborates, “the level of rationalization of a religion is suggested in two ways: negatively, by the degree to which magical elements are eliminated and positively, by the degree to which its ideas and its relations to the world are systematically unified.” The totally different dynamic of economic and political rationalization however “is seen most clearly in the ubiquitous growth of bureaucracy” (Hughey, 1979, p. 103). But it is not clear how this difference in modes of rationalization between the religious sphere and the political and economic spheres would generate any tension or how and why such a tension would lead to a decline of religiosity or the social significance of religion. One might even surmise that they would support each other. Indeed, modern “secularism” has been a reasonable mode of accommodation whereby the state is independent of religion(s) while religiosity thrives through freedom of belief in society. Curiously, this does not appear among Weber's “solutions” to the tension.

## Marx on reifying abstractions

According to Karl Marx, the political arrangement just described implies “the emancipation of the state” from religion, but is an unsatisfactory arrangement: “To be *politically* emancipated from religion … is not the final and absolute form of *human* emancipation” (Marx, 1843, p. 32). Whether the “final and absolute form” will ever take place may be left aside as an open question. Regarding the question of “secularization,” however, it is clear that “political emancipation from religion,” designed as a political formula for governing religious diversity in society by stripping the centralized state of a religious identity, has little to do with the “decline of religion” as per the modernization theory.

Marx's ideas on religion have had a tremendous impact, although he never wrote on the topic systematically. They were scattered over his writings and occasionally pieced together by later scholars (Achcar, 2020; Toscano, 2010; McKinnon, 2005). We may begin our search for a theory of secularization with his famous metaphor for religion as the “opium of the people” (Marx, 1844, p. 54). If we consider the identity of the people who were ingesting this “opium” at the time and place in which he was writing, it will be obvious that he meant the working class, the downtrodden dwellers of modern capitalist society. For Marx, then, capitalist modernity did not drive out religion. If anything, it made religion perhaps even more relevant to the lives of the working masses, because their conditions of exploitation were hidden from view, forcing them to lean on the “sentiment of a heartless world,” in terms of another metaphor for religion that Marx uses in the same paragraph.

So understood, Marx's notion has been vindicated on at least two historical occasions: First, the rise of Protestantism as a form of religious fundamentalism that accompanied the transition to capitalism; and second, the recent global spread of religious fundamentalisms following the end of the Cold War that accompanied the decline of communism in the East and of the welfare state in the West, both of which provided some form of socio-economic shelter to the working classes. The global spread of neoliberalism thus went in tandem with a wave of de-secularization. This was to be expected because, as Marx explains, religion serves a function: “*Religious* suffering is at the same time an *expression* of real suffering and a *protest* against real suffering” (Marx, 1844, p. 54). It is a refuge that helps people suffer the insufferable (cf. McKinnon, 2005).

The sharpest exposition of Marx's theory of religion, however, can be found not in the just-cited two sources (Marx, 1843, 1844), but in what may seem an unlikely source, *Capital, Vol. I* (1867), in which Marx draws parallels between religion and capitalism. He explicates what he calls the “fetishism” of commodities in capitalism by reference to his conception of religion. While describing the commodity as “a mysterious thing,” which hides the “definite social relation between men, that assumes, in their eyes, the fantastic form of a relation between things,” he suggests that “to find an analogy, we must have recourse to the mist-enveloped regions of the religious world. In that world the productions of the human brain appear as independent beings endowed with life, and entering into relation both with one another and the human race” (Marx, 1974, p. 77). Religion and the commodity (and its value-form, *money*) are both products of reified abstractions, which then turn around to dominate the human subject: In the realm of capitalist production, the laborer “constantly produces material, objective wealth, but in the form of capital, of an alien power that dominates and exploits him” (Marx, 1974, p. 535). Thus: “As, in religion, man is governed by the products of his own brain, so in capitalistic production, he is governed by the products of his own hand” (Marx, 1974, p. 582).

According to Marx, deposing both abstractions will take place simultaneously. Marx clarifies this in remarks that follow his allusion to the “opium” metaphor, which in isolation is usually mistaken as a pejorative description: “The abolition of religion as the *illusory* happiness of men is a demand for their *real* happiness. The call to abandon their illusions about their condition is a *call to abandon a condition which requires illusions*.” (Marx, 1844, p. 54). In other words, as long as the conditions that create this illusion persist, the illusion itself will remain. Marx also affirms this in *The Manifesto*: “the social consciousness of past ages… cannot completely vanish except with the total disappearance of class antagonisms” (Marx and Engels, 1848, p. 489). He explains why this should be so in *Capital*: “The religious world is but the reflex of the real world. … The religious reflex of the real world can, in any case, only then finally vanish, when the practical relations of every-day life offer to man none but perfectly intelligible and reasonable relations with regard to his fellowmen and to Nature.” (Marx, 1974, p. 83–84).

If the existing social conditions, that is, capitalism and the associated social relations of domination and exploitation, generate the religious illusion, it does not serve to just criticize the illusion; the underlying conditions need to be removed, upon which the religious illusion will also disappear. As Owen Chadwick accurately perceives, while some Marxists fail to see (cf., however, Boer, 2009), it does not make sense for Marx to struggle against religion. Chadwick confirms Marx's insight by noting that although church attendance declined in England in the late 19^th^ century, it was not because atheism was popular among the working class, as is often thought. He points out that atheists and secularists “were always a small minority... their activities hardly touched the mass of the population.” (Chadwick, 1975, p. 89).

Although not widely recognized, there is much in common between Marx's and Durkheim's perspectives on religion. They diverge, however, on the possibility of total “emancipation” from it. Like Marx, Durkheim too thinks of religion as the reification of an abstraction, though he expresses it differently; but he also thinks that religion is unlikely to ever disappear. The difference lies in their respective concepts of the source of people's consciousness. For Marx, social consciousness derives from class position; whereas for Durkheim consciousness comes from society at large. Thus, even if, as Marx claims, the collective belief system that illusorily soothes the suffering of the working class disappears when class conflict ends, Durkheim argues that another collective set of ideas will take its place as long as society remains a structured entity. From Durkheim's point of view, what Marx considers the final “*human* emancipation” from religion is nothing but the establishment of yet another set of sacred norms and collective sentiments.

## Durkheim on deifying society

According to Pickering (1984, p. 442), secularization was a major theme for Emile Durkheim, who considered it to be an ongoing process throughout history and did so “in far more radical terms than do many modern sociologists.” But Pickering adds, “Nevertheless, he had a paradoxical approach to the subject: religion is dying and society is becoming secular; yet, on the other hand, religion is alive and will always be a component of social life.”

This is a surprising interpretation. In fact, there was no paradox: Durkheim simply changed his view. In his first book, *The Division of Labor in Society* (1893), Durkheim confidently asserts, “if there is one truth that history has incontrovertibly settled, it is that religion extends over an ever diminishing area of social life” (Durkheim, 1984, p. 119). In his last book, *The Elementary Forms of Religious Life* (1912), however, he thinks differently: “There is something eternal in religion… No society can exist that does not feel the need … to sustain and reaffirm the collective feelings and ideas that constitute its unity and personality” (Durkheim, 2001, p. 322).

Durkheim's first book (*DoL*) contains only a few pages of cursory remarks on religion, prefaced with the observation that “At the present time we do not possess any scientific conception of what religion is” (Durkheim, 1984, p. 118), probably implying that it would be his task to accomplish that. Indeed, his last book (*EFoRL*) is completely devoted to the topic and should be considered his final word on the matter. Also, unlike Weber, there is no ambiguity in Durkheim's ideas. However controversial (Pickering, 2001; Koenig, 2024), his final theory of religion is rather straightforward. Judging by this work, one could not ascribe a secularization thesis to Durkheim.

In *DoL*, Durkheim famously distinguishes between “mechanical” and “organic” social solidarity. In the former type, characterizing traditional society, a common morality holds society together. In modern industrial society, however, this “common consciousness” declines as individual autonomy develops. Under these conditions, he suggests, “social life proper must either diminish or another form of solidarity must emerge gradually to take the place of the one that is disappearing,” and adds, “It is the division of labor that is increasingly fulfilling the role that once fell to the common consciousness” (Durkheim, 1984, p.122, p.123). In this context, Durkheim clearly endorses a secularization theory. But then the notion of “mechanical v. organic solidarity” does not appear in any of his subsequent work (Lukes, 2013, p. xxviii). Even in his book on *Suicide* (1897), where the modes and degrees of solidarity are treated as determinants of rates of suicide, this classification is not cited.

Before reaching his mature theory of religion, Durkheim experiments with some other ideas left undeveloped. A prominent one, revealing the central question that leads him to his mature theory, is the concept that has been popularized in secondary literature as the “cult of man,” “cult of individualism,” or “religion of humanity.” Already in *DoL*, apparently uncomfortable with the idea that division of labor alone would sustain solidarity, and assuming that some kind of common morality would still be necessary, he considers the notion that a shared mutual respect for individual rights characterizes modern society. While describing the decline of common consciousness as an “iron law,” he also warns that “this is not to say that the common consciousness is threatened with total disappearance.” He notes the existence of “indeed one area in which the common consciousness has grown stronger, … viz., in its view of the individual. … We carry on the worship of the dignity of the human person, which like all strong acts of worship, has already acquired its superstitions. If you like, therefore it is indeed a common faith.” (Durkheim, 1984, p. 122). Durkheim revisits this concept in *Suicide*, while discussing what he calls *egoistic* suicide: “in societies and environments where the dignity of the person is the supreme end of conduct, … the individual is readily inclined to consider the man in himself as a God and to regard himself as the object of his own cult.” (Durkheim, 2002, p. 331).

This concept has been stretched in various directions by commentators. Paul Carls, for example, suggests that “Durkheim's formulation of the cult of the individual anticipated later discussions of democratic theory,” such as John Rawls' “overlapping consensus” and the German concept of “constitutional patriotism” (Carls, 2019, p. 3). More convincingly, Paul Heelas analyzes the “New Age” by reference to Durkheim's concept: “the New Age appeals to (relatively) detraditionalized selves, who are seeking autonomous self-cultivation, aspiring to ground their identity within, wanting to exercise their independence, authority, choice and expressivity” (Heelas, 1996, p. 162, *passim*).

But it is highly unlikely that Durkheim himself would endorse these interpretations. For him, it was a tentative idea that he eventually abandoned. Already in *DoL*, as soon as he raises this idea, he adds the caveat that even “if the faith is common because it is shared among the community, it is individual in its object. … Thus it does not constitute a truly social link.” (Durkheim, 1984, p. 122). This last point is important, because Durkheim defines “religion” by this criterion, as we see below. Regarding his citation of this “cult” in *Suicide*, he clearly does not ascribe to it the positive value implied in the secondary literature. He says: “When morality consists primarily in giving one a very high idea of one's self, … man [may become] unable to perceive anything above himself. … Thus, egoistic suicide arises.” (Durkheim, 2002, p. 331).

Wallwork (1985, p. 203) argues that Durkheim's early work contains the seeds of his mature theory of religion: “Society, conceived as a superior spiritual or psychical phenomena [*sic*], is viewedfrom the very outset of Durkheim's career as the true source of the experience of transcendent authority.” The short-lived “cult of individualism” thesis may be seen in this context. Observing the rise of individualism, but also thinking that a common morality is still necessary, Durkheim seems to have found this idea, which he also recognized was internally contradictory (because this faith “does not constitute a truly social link”) and eventually abandoned along with his concept of “mechanical v. organic solidarity.” In his last book (*EFoRL*) Durkheim no longer speaks of the autonomy of the individual and thinks that a common consciousness still prevails (or must prevail), perhaps with a new set of norms, beliefs, and values, replacing traditional consciousness. Still, in *EFoRL*, he aims to answer questions already posed in his first book. For instance, in *DoL*, referring to “the notion of God,” he says, “it remains to be explained how men have been led to ascribe such an authority to a being who, on the admission of everybody, is in many, if not all cases, a figment of their imagination” and ponders: “the force that the being possesses must come from somewhere” (Durkheim, 1984, p. 119). The explanation appears in *EFoRL*.

Durkheim's answer to his own question contrasts with Weber's. First, he thinks that religion does not derive from magic; it is rather the other way around (Durkheim, 2001, p. 269). This is because magic is utilitarian and individualistic, whereas religion is “eminently collective” and involves a system of morality. Unlike religion, there is no “church of magic” and “no sin in magic” (Durkheim, 2001, p. 43–46, p. 223). Second, religion does not originate from the fear of natural forces or a belief in the supernatural. We saw above that Weber is somewhat ambiguous on this point, leading some to interpret his concept of “disenchantment” as an indicator of “secularization.” Durkheim, however, is very clear: “if expressing the forces of nature is the chief purpose of religion, it is impossible to see religion as anything but a system of misleading fictions whose survival is incomprehensible” (Durkheim, 2001, p. 71, p. 169–171). Religion does not aim to explain reality; it represents society, the individual's attachment to it and to its moral norms, which is why it is “eternal.” Durkheim builds this argument through an analysis of the meaning of the “totem” in his study of early religions. “On the one hand,” he says, the totem “is the external and tangible form” that symbolizes the god of the clan. “But on the other, it is the symbol of that … clan. … So if the totem is both the symbol of god and society, are these not one and the same? … The god of the clan … must therefore be the clan itself, but transfigured and imagined in the physical form of the [totem]” (Durkheim, 2001, p. 154).

The similarity here with Marx's analysis is unmistakable, although Durkheim never cites Marx and is keen to distance himself from “historical materialism” (Durkheim, 2001, p. 318–319). Durkheim's concept of “imagination” (or “transfiguration”) resembles Marx's description of religion as a force created in our minds by us and yet dominates us. Durkheim (somewhat misleadingly) emphasizes throughout the book that religion is not “illusory,” meaning that it is real in people's lives and has a concrete social foundation: “there are no false religions … all respond … to the given conditions of human existence” (Durkheim, 2001, p. 4, *passim*). But apart from the way the foundation is explained, this idea is identical to Marx's.

Steven Lukes's interpretation of Durkheim's theory of religion implicitly contradicts this point. Lukes (2012, p. 43) observes that Durkheim was concerned whether his theory might be seen as “subversive of the beliefs of the faithful” and intent on making sure that it was not: “Indeed, he remarked, a rational interpretation of religion could not be thoroughly irreligious, since an irreligious interpretation would deny the very fact it sought to explain.” But aside from a possible expression of respect to the faithful, which did not really concern Marx, Durkheim's explicit goal is to subject religion as a “social fact” to scientific examination, which goal he obviously shares with Marx and which would indeed be meaningless if people did not have faith in *something*, even if it were the wrong thing.

Durkheim was personally non-religious (Lukes describes him as “laic”) and Marx, despite his assertive atheism, urged a struggle not against religion, but against the conditions that generated it as “false consciousness.” Durkheim, on the other hand, similar to Marx's allusion to the “commodity” as a reified abstraction, offers the following analogy to explain religion. Describing the flag of the nation as a modern totem, he explains: “The soldier who dies for his flag, dies for his country; but in his mind, the flag comes first. … the flag is only a sign, … it has no intrinsic value but serves only to recall the reality it represents; we treat it as if it were that reality” (Durkheim, 2001, p. 165). He further elaborates, “Sacred beings exist only because they are imagined as such. If we cease to believe in them, they will cease to exist” and “while superior to men, [they] can live only in human consciousness” (Durkheim, 2001, p. 256, p. 257). According to Durkheim, “A society is the most powerful bundle of physical and moral forces observable in nature” (Durkheim, 2001, p. 342). Religion for Durkheim, then, is the deification of society and the totem is the reification of society's power.

## Durkheim v. Marx

Along with the similarities, there are also significant differences between Marx's and Durkheim's theories of religion. For Marx “human emancipation from religion,” though not achieved under capitalism, is both possible and desirable. It is predicted to occur after a proletarian revolution when religion and the state, both of which currently serve to maintain class divisions, will “wither away.” For Durkheim, however, secularization is neither possible nor desirable. We have seen that this difference originates from their respective conceptions of social consciousness. For Marx it is based on class position; for Durkheim, on the collective morals of society. Durkheim thinks that there will be religion as long as there is society and hence the need for social solidarity. It follows, in this reasoning, that if and when the social system desired/predicted by Marx is created, there will necessarily be a new collective moral order. By implication, although Durkheim never says so explicitly, the ideological current advocating this new moral order (i.e., Marxism) is a type of religion. In fact, Durkheim explicitly describes a comparable episode, the immediate aftermath of the French Revolution, as the creation of a new religion. This reference reveals yet another difference from Marx. Durkheim underscores collective “rituals” as a central element of religion and religiosity, while Marx totally ignores them.

According to Durkheim, rituals form the essential link between the individual and collective consciousness: “acts of worship … are not futile or meaningless gestures. By seeming to strengthen the ties between the worshiper and his god, they really strengthen the ties that bind the individual to his society, since god is merely the symbolic expression of society.” (Durkheim, 2001, p. 171). They are also essential for the very existence of religion: “Certainly, without the gods, men could not live. But on the other hand, the gods would die if the cult were not celebrated” (Durkheim, 2001, p. 256). Thus, representations and rites are the two pillars of Durkheim's theory of religion: collectively created and worshiped in rites, sacred symbols cement society. He interprets the French Revolution within this conceptual framework: “Society's capacity to set itself up as a god or to create gods was nowhere more visible than in the first years of the Revolution. … A religion propelled by its own momentum was established with its dogma, symbols, altars, and holidays. The cult of Reason and of the Supreme Being tried to bring a kind of official fulfillment to these spontaneous aspirations” (Durkheim, 2001, p. 161). He adds that the experience was transitory, but still instructive: “it is these effervescent social settings, and from this very effervescence, that the religious idea seems to be born” (Durkheim, 2001, p. 164).

Numerous authors both before and after Durkheim have found aspects of religious fervor in the French Revolution, though for different reasons. Alexis de Tocqueville argued in 1858 that, seemingly anti-religious, the French Revolution was actually similar to a religious revolution: “like all great religious movements it … broadcast a gospel … [and] sought proselytes all over the world” (De Tocqueville, 1995, p. 11). He further notes: “The chief aim of a religion is to regulate both the relations of the individual man with his Maker and his rights and duties toward his fellow men on a universal plane”, and, like a religion, the Revolution sought to reach beyond French borders “to determine the rights and duties of men in general toward each other and as members of a body politic” (De Tocqueville, 1995, p. 11, p. 12). Still, he ends the discussion with some qualification: “It would perhaps be truer to say that it developed into a species of religion, if a singularly imperfect one, since it was without a God, without ritual or promise of a future life” (De Tocqueville, 1995, p. 13). Two elements emerge in de Tocqueville's conception of religion: the institution of a new moral order (which agrees with Durkheim's) and the universalizing mission (which does not). But then in his qualification de Tocqueville lists further characteristics (God, ritual, and promise), the significance of which, or even their absence in the Revolution, Durkheim would dispute.

The ritual element is central in the analysis of Zerubavel (1985, p. 28–34), who notes that the French Revolution introduced several cultural novelties in an effort to oppose Christian tradition and conform to the “Age of Reason.” Among them was the institution of a new calendar with standard thirty-day months divided into three 10-day weeks and the division of the day into 10 h, each of which had 100 decimal minutes. These were turned into rules, strictly enforced through penalties on people who went to church on the traditional Sunday and did not close their shops on the revolutionary weekend. But the experiment was short-lived as the rules were widely defied and soon officially abandoned.

De Tocqueville's identification of religion with a universalizing mission indirectly challenges Durkheim's theory. De Tocqueville underlines the significance of this feature by contrasting it with the “pagan religions of antiquity” which reflected the “social order of their environment, … [and which] functioned within the limits of a given country and rarely spread beyond its frontiers” (De Tocqueville, 1995, p. 12). But it was precisely this type of “religion” that formed the empirical material for Durkheim's theory, apparently trapping him into equating specific religions with specific societies. The theory would thus imply that no two societies could have the same god/religion, each society would only have one god/religion, and that social change would generate a new god/religion. As none of these contentions can be sustained, Durkheim's theory cannot account for the variety of societies that have shared the tenets of a given universal religion and have done so in different historical periods. Society cannot be worshiping itself if innumerable societies across time, space, and social conditions have shared the same religious symbols and if a given society contains more than one religion.

Durkheim is not unaware of these problems. He notes, though only in passing, that “religious beliefs display a tendency to resist enclosure in a politically delimited society,” and so briefly cites “religious universalism” (Durkheim, 2001, p. 320–321). Regarding social and religious change, a redeeming remark appears in an unrelated, earlier book (of 1895): “The religious dogmas of Christianity have not changed for centuries, but the role they play in our modern societies is no longer the same as in the Middle Ages. Thus words serve to express new ideas without their contexture changing” (Durkheim, 1982, p. 121).

A large literature treats nationalism as the functional equivalent of religion (Santiago, 2012), apparently inspired by Durkheim's theory but also adding elements to it. Llobera (1994, p. 143) argues that nationalism is “a secular religion where god is the nation. … [With] all the trappings and rituals of a religion, … it has [also] tapped into the emotional reservoir of human beings.” Marvin and Ingle (1999) combine Durkheim's description of the national flag as a totem with their own conception of “sacrifice” as a central element of religion. They argue that “civil religion and nationalism are synonymous terms for the sacralized agreement that creates killing authority and specifies the relationship of group members to sacrificial death” (Marvin and Ingle, 1999, p. 11). While some authors imply that nationalism displaces religion, others find a continuity or synthesis between them (Van der Veer and Lehmann, 1999; Marx, 2003; Smith, 2003). Finally, some go beyond nationalism to link the concept of god or religion with state power more generally (Lane, 1981; Nicholls, 1994). All these reflections seriously challenge secularization theory, but are still often framed in terms of a concept of “secular religion” or “quasi-religion.” From Durkheim's point of view, however, they are just *religions*.

Those who speak of “quasi-religions” have a narrower concept of religion than Durkheim's. Their typical point of reference is Christianity and specifically its promise of salvation in life after death (which, though often elided, is shared by the other two Abrahamic traditions). According to Smith (1994, p. 3), for example, quasi-religions are similar to “religions proper,” as they *diagnose* the human condition as a predicament, offer a *quest* for its solution, and a *deliverer* to overcome the flaw and bring salvation. But they are also significantly different because they have “no place for a transcendent reality”; instead, they absolutize “a particular reality that is finite and conditioned … as the object of loyalty” (Smith, 1994, p. 7–8). Smith examines Humanism, Marxism, and Nationalism as quasi-religions, and identifies their objects of loyalty, respectively, as humanity, the working-class, and the nation-state. Durkheim's more expansive definition of religion, however, makes no mention of a promise of salvation: “a religion is a unified system of beliefs and practices relative to sacred things, that is to say, things set apart and surrounded by prohibitions—beliefs and practices that unite its adherents in a single community called a church.” (Durkheim, 2001, p. 46). This definition is broad enough to include “secular” ideologies, which are obviously produced by humans, and implies that religions have the same provenance.

## Rethinking secularization theory

The arguments of this paper engender some new questions, which could not be pursued here but might become subjects for further research. The most obvious one, interesting from the perspective of the history of sociological thought, regards the conditions under which these classical authors were retrospectively misdescribed as secularization theorists. This was likely a product of the postwar era in triumphant United States, engaged in the Cold War, an era when modernization theory reigned supreme, positing that modernity was an inevitable (*and* desirable) destination, and secularization and democracy were its necessary consequences. The decline of modernization theory, along with the end of the Cold War, gave rise to the (temporary) hegemony of postmodernism. Two examples that bracket this period at either end, each from a different part of the non-Western world, may help illustrate this point. First, the concept of separation of church and state was written into the Japanese constitution by the Allied Occupation at the end of World War II (Hardacre, 1989; Yamagishi, 2008), implying that if secularization does not take place spontaneously through domestic social change, then it ought to be imposed from outside. Second, postmodernism and multiculturalism in the West arose concurrently with Islamism in Turkey (and elsewhere), with remarkable and perhaps surprising intellectual parallels between them (Gülalp, 1995, 1997; see also Afary and Anderson, 2005). Why the misdescription about our authors has persisted, however, is not very clear, but may be due to the fact they all were modernist and secular (non-religious).

Another question concerns Durkheim's more expansive concept of religion than the traditional one that underlies the secularization thesis. His theory offers a vision for how a decline in traditional religious activities may be replaced by a growing adherence to “quasi-religious” currents, thus leaving religiosity in general intact. But, as noted, the theory is not problem-free. Perhaps Durkheim could have advanced his theory by incorporating a concept proposed by Jean-Jacques Rousseau in *The Social Contract* (1762). Somewhat short on coherence, and motivated by a hostility ambiguously toward Christianity in general or the Church in particular, Rousseau names three types of religion, the “religion of man,” the “religion of the priest,” and the “religion of the citizen” or “civil religion,” and then, concerned like Durkheim with the necessity of social solidarity and judging the first two types lacking in this regard, he advocates the “civil religion,” but does so with a curious twist. Having found some elements of “evil” in its original version, typical in “early nations,” he updates it by suggesting that there is “a purely civil profession of faith, the articles of which it is the duty of the sovereign to determine,” and details his vision: “The dogmas of civil religion ought to be simple, few in number, stated with precision, and without explanations or commentaries” (Rousseau, 1998, p. 137). Religious diversity (in the traditional sense) may still be achieved under a single “civil religion”: “Now that there is, and can be, no longer any exclusive national religion, we should tolerate all those which tolerate others, so far as their dogmas have nothing contrary to the duties of a citizen” (Rousseau, 1998, p. 138). Durkheim does not seem to have considered how Rousseau's concept could help revise his own ideas to allow for the possibility of having several religions in one society, although their ideas clearly overlap. A synthesis was later offered by Bellah (1967), who described American patriotism as a “civil religion.” It is different from Christianity; therefore, it is possible for citizens to follow both a traditional and the civil religion.

Our discussion also indicates a way in which secularization might be disaggregated into its distinct components. First, *political* secularization (Marx's “emancipation of the state from religion”) has indeed taken place historically and remains a desired goal normatively, but it can be and has been reversed in recent decades. Casanova (1994) describes this process as the “deprivatization” of religion, i.e., the reversal of the privatization previously observed and theorized. Second, evidently, political secularization does not necessarily lead to *social* secularization (i.e., declining social significance of religion). On the contrary, it allows for the flourishing of various belief systems within civil society. No doubt, the rhythms of modern (particularly economic) life make it harder to regularly attend or get involved in organized institutional religion; but in its place arise new trends of believing, such as what scholars have called “implicit religion,” “private spiritualism,” or “believing without belonging” (Davie, 1990), leaving aside quasi-religions altogether. Third, considering the prevalence of quasi-religions alongside the traditional ones, in addition to new forms of spirituality, a third aspect of secularization, *intellectual* secularization, described by (Kant 1784) in his exhortation, “*sapere aude*” *(“dare to think for yourself”*), appears hard to come by and is indeed ruled out by both Marx and Durkheim. In their sociologies, consciousness is ultimately constrained by the norms and structures of society, which individuals cannot transcend.

The modern individual is a not a natural constituent of society, it is rather its product. Individualism itself is an outcome of social structures. According to Durkheim (1984, p. 220–21), “Collective life did not arise from individual life; on the contrary, it is the latter that emerged from the former.” In the words of (Marx 1859, p. 4), “It is not the consciousness of men that determines their being, but, on the contrary, their social being that determines their consciousness.” Marx predicts that class divisions that form the currently prevailing consciousness will eventually end and freedom of the intellect will begin (which he also describes as the “end of prehistory”). For Durkheim, however, that will never occur because without a common consciousness that holds individuals together society would fall apart. He explains: “Even today, with all the freedom we grant each other … there is a principle that even peoples most enamored of free enquiry tend to place above discussion and to regard as untouchable, or sacred: that is the principle of free enquiry.” (Durkheim, 2001, p. 161). The difference between these two predictions originates from a fundamental difference between their assumptions. Marx's “Theses on Feuerbach” includes the following critique: “Feuerbach resolves the religious essence into the human. But the human essence is no abstraction inherent in each single individual. In its reality it is the ensemble of the social relations. … Feuerbach consequently does not see that the ‘religious sentiment' is itself a social product, and that the abstract individual belongs in reality to a particular form of society.” (Marx, 1845, p. 145). But that is precisely what Durkheim does as well. He too “resolves the religious essence into the human.” This brings us to our final question, briefly formulated below, that deserves further research.

## Conclusion: nature v. nurture?

Clark (2012) reviews a wide range of historical and theoretical literature to demonstrate that religiosity has not declined in any meaningful sense over the course of the centuries during which “modernization” is supposed to have taken place in the Western world (or that at least there is no clear evidence one way or the other) and thus offers a detailed analysis of the historical myths on which secularization theory is based. Owen Chadwick wonders whether the “religious attitude to the world” persisted “because it had roots in *human nature*” (Chadwick, 1975, p. 263, *italics added*). Durkheim describes his own project in *EFoRL* as an attempt to understand “the religious *nature of man*, … an essential and permanent aspect of humanity” (Durkheim, 2001, p. 3, *italics added*).

Durkheim's analysis of religion as a social fact derives from a basic observation, although he never puts it in these terms: Religious beliefs and identities among human societies have been diverse and variable throughout history, often in competition and even in conflict with each other, but the experience of believing itself is commonly shared across humankind. How do we account for this? Culture cannot be the answer, since there is no unified human culture. But there must be something in the nature of human beings, where the answer may be sought.

Observing the universality and permanence of certain human traits, despite the geographical variety and historical variability of cultures and social structures, raises the question of the possible existence of some “natural” tendency among human societies, as opposed to “nurture,” to invoke the typical juxtaposition. This question has generated much controversy, albeit with a growing acceptance among sociologists that there might indeed be evolutionary processes, both neurobiological and social psychological, that shape human behavior along the lines of adaptive advantage. It is certainly a question worth pursuing.
